# Comparative effects of Bowen therapy and tennis ball technique on pain and functional disability in patients with thoracic myofascial pain syndrome

**DOI:** 10.1186/s13018-023-04379-z

**Published:** 2023-11-24

**Authors:** Fareeha Amjad, Afsheen Khalid

**Affiliations:** Department of Physical Therapy, Riphah International University, Lahore, Pakistan

**Keywords:** Myofascial pain syndrome, Myofascial trigger points, Pain, Disability

## Abstract

**Background:**

Thoracic myofascial pain syndrome is a clinical problem arising from the muscles and soft tissues of thoracic region, which include the mid and upper back area. Risk factors associated with myofascial pain syndrome are muscle overuse and repetitive strain, poor posture, trauma or injury, emotional and psychological stresses. The management of myofascial pain syndrome (MPS) typically involves a multidimensional approach that focuses on relieving pain, reducing muscle tension, and improving muscle function. Bowen therapy and tennis ball technique are also recommended for treating myofascial pain syndrome.

**Objective:**

The objective of this study was to compare the effects of Bowen therapy and tennis ball technique on pain and functional disability in patients with thoracic myofascial pain syndrome.

**Methods:**

It was a randomized clinical trial conducted on thirty patients. It was carried out in physiotherapy outpatient department of D.H.Q Hospital, Kasur. Non-probability convenience sampling technique was used. Data collection was done from the patients of thoracic myofascial pain syndrome by using Numeric Pain Rating Scale (NPRS) for pain and Pain Disability Questionnaire (PDQ) for functional disability. Participants were randomly allocated into two groups using computer generated random number method. Group A received Bowen therapy, and group B received tennis ball technique. Outcome measures were measured at baseline, after second week treatment session and after fourth week with three sessions in a week on alternate days. Data analysis was done by using Statistical Package for the Social Sciences (SPSS) version 26.

**Results:**

There was significant difference between the mean values of NPRS and PDQ in both groups at baseline, second week and fourth week with *p* value < 0.05. The results indicated that both treatments were significant but Bowen therapy is more effective treatment than tennis ball technique. Within-group difference calculated with repeated-measure ANOVA indicated that there was significant difference from pre- to post-values of both groups**.**

**Conclusion:**

This study concluded that Bowen therapy produced statistically significant and clinically relavant results for all the outcome measures. *Trial registration*. (IRCT20190717044238N7).

## Background

Myofascial pain syndrome (MPS) is a disorder that causes pain and discomfort in the muscles and surrounding soft tissue. It is caused by the existence of trigger points, which are sore areas in the muscle that become painful when pressure is applied [1]. These trigger points are tight bands or knots of skeletal muscle fibers that can be palpated and are tender to touch. They are associated with local pain as well as referred pain patterns [2]. Thoracic myofascial pain syndrome is a clinical problem arising from the muscles and soft tissues of thoracic region, which include the mid and upper back area. The thoracic region comprises numerous muscles, including the erector spinae, rhomboids, trapezius, and intercostal muscles [3]. Common symptoms of thoracic myofascial pain syndrome include localized pain, referred pain, trigger or tender points [4]. The occurrence of MPS in the overall population has been reported up to 85%. In clinical settings, the prevalence of MPS can vary depending on the specific patient population. Many studies have been done on prevalence of MPS in different population. Some studies have described prevalence rates of MPS ranging from 30 to 93% in numerous clinical populations, such as primary care, pain health center, and specialty clinics concentrating on musculoskeletal disorders [5]. The pathophysiology of myofascial pain syndrome (MPS) is not that much clear and is the question of continuing research. However, quite a lot of mechanisms have been suggested that add to the development and persistence of myofascial trigger points and related pain [6]. The hallmark symptom of MPS is musculoskeletal pain which is local or regional pain in the muscles or soft tissues [7]. The measures for diagnosing myofascial pain syndrome (MPS) are primarily based on therapeutic assessment and the presence of characteristic features. Physical examination plays important role in diagnosis [8].

One commonly used set of criteria is the "Diagnostic Criteria for Myofascial Trigger Points" developed by Simons et al. They proposed five major and three minor criteria. Here are the diagnostic criteria for MTrPs according to Simons et al. The major criteria include (a) regional and impulsive pain; (b) palpable taut band in accessible muscle; (c) within the taut band, there should be a tender point, exquisite localized tenderness; (d) the palpation of tender point should reproduce or intensify the patients typical complaint of pain; and (e) decreased range of motion, when measurable. The minor criteria include (a) altered sensation or propagation of pain complaint due to tender points; (b) local twitch response by inserting needle into the taut band; and (c) pain is diminished or decreased by muscular therapy (stretching of muscles or injection of the MTrPs).

Presence of five major and one out of three minor criteria is necessary for diagnosing myofascial pain syndrome [9].The management of myofascial pain syndrome (MPS) usually involves a multidimensional approach that focuses on improving pain complaint, decreasing muscle tension, and improving muscle function. A noninvasive treatment is extracorporeal shock wave therapy (ESWT) that has been used for several musculoskeletal disorders, including myofascial pain syndrome. In this treatment, high-energy sound waves are used that target the affected area [10]. Ischemic compression, spray and stretch, muscular energy techniques, transverse friction massage, ultrasound therapy, thermotherapy, and dry needling are some of the therapeutic methods used to reduce trigger point discomfort [11]. Myofascial release is a manual therapy technique used to address restrictions and tension that present in the fascia. Muscles, bones, and organs are surrounded by connective tissue sheath called fascia. It aims to release tightness, increase mobility, and reduce pain by applying sustained pressure. Pressure can be applied to the affected area using hands, fingers, or specialized tools. Bowen technique or Bowen therapy (BT) is a holistic bodywork technique developed by the late Tom Bowen in the 1950s in Australia. It is a gentle and noninvasive approach that aims to promote healing, pain relief, and overall well-being [12]. The Bowen technique is based on the principle that these gentle movements can prompt the body's innate healing mechanisms, encouraging self-regulation and balance. The practitioner uses their hands to make subtle, rolling movements over specific points on the body, applying gentle pressure to stimulate the fascia [13]. Another treatment approach is myofascial release technique using a tennis ball. It is a form of self-massage that can be used to target trigger points and release tension in the muscles and fascia. Using a tennis ball for myofascial release can provide temporary relief of muscle tension and promote relaxation [14]. This study aimed to compare the effects of Bowen therapy and tennis ball technique on pain and function disability in patients with thoracic myofascial pain syndrome.

## Methodology

### Study design

The study was randomized clinical trial. Trial was registered in IRCT with IRCT reference number: IRCT20190717044238N7.

### Study setting

Data were collected from the physiotherapy department of D.H.Q Hospital, Kasur.

### Procedure

A single blinded, randomized clinical trial was used in this investigation, which was lasted for ten months. In this study, 30 patients with thoracic myofascial pain syndrome were selected after meeting the inclusion and exclusion criteria. According to inclusion criteria, participants were included on the basis of diagnostic criteria explained by Simon and Travell et al. for MPS: the existence of five major (regional and spontaneous pain, palpable taut band, tenderness over the taut band, referred pain, decreased range of motion) and at least one out of three minor signs (pain on pressure, local twitch response, decrease in pain) [15] and age between 18 and 40 years [16]. Male and female patients were included. Patients with fibromyalgia and any other deformity like scoliosis and those presenting signs of any skin disease were excluded. The Institutional Review Board (IRB) of Riphah International University in Pakistan officially authorized the study before enrolling patients who met the inclusion criteria and provided written informed permission. A record of the demographic information, including height, weight, and body mass index (BMI) was made. Prior to the therapy, a consent form was filled out. Two groups were made. Group A received Bowen therapy, and Group B received tennis ball technique. Results were evaluated at baseline, after second week and at the end of fourth week. The data were analyzed using SPSS version 26.

### Outcome measures

#### Numeric Pain Rating Scale (NPRS)

This scale is used for assessing pain. English version of NPRS was used in this study. Total score is 0–10. With 0 representing ‘no pain’ and 10 representing ‘pain at its extreme,’ the patients were requested to select a number between 0 and 10 that best described their pain before and after treatment. NPRS declared the good test-retest reliability is r = 0.79–0.96 (ICC = 0.94; 95% CI 0.61–0.96) [17].

#### Pain disability questionnaire (PDQ)

The Pain Disability Questionnaire (PDQ) is tool for assessing disability caused by pain. English version of this scale was used. The total score of PDQ is 0–150. The following ranges are used to examine the score: mild/moderate (0–70), severe (71–100), extreme (101–150) [18]. PDQ exhibited good reliability level (ICC = 0.87), validity = 0.62, and Cronbach’s alpha = 0.69 [19].

#### Randomization

Using computer software (https://www.randomizer.org/), subjects were divided into two groups at random. The allocation list was created using the approach of sealed, opaque envelops, labeled 1 for group A and 2 for group B. Thirty participants were divided into two groups fifteen in each group. Group A participants received Bowen therapy, and group B participants received tennis ball technique. The CONSORT flow diagram represented the process of participants’ assignment to these groups.

#### Intervention

##### Group A (Bowen therapy)

Patients were asked to wear light, loose-fitting clothing. Patients were prone lying. While applying Bowen technique, fingers were used to apply gentle, rolling movements on both sides of thoracic region along the medial border of scapula. Gentle pressure for thirty seconds was applied. Each treatment session lasted for 15–20 min with two–three repetitions. The skin was stretched and moved with every rolling movement. The movements were repeated after 2 min of rest interval as necessary [20]. Three treatment sessions were given in a week for total four weeks. Mid-treatment assessment was done on second week, and follow-up assessment was on fourth week.

##### Group B (Tennis ball technique)

**Fig. 1:**
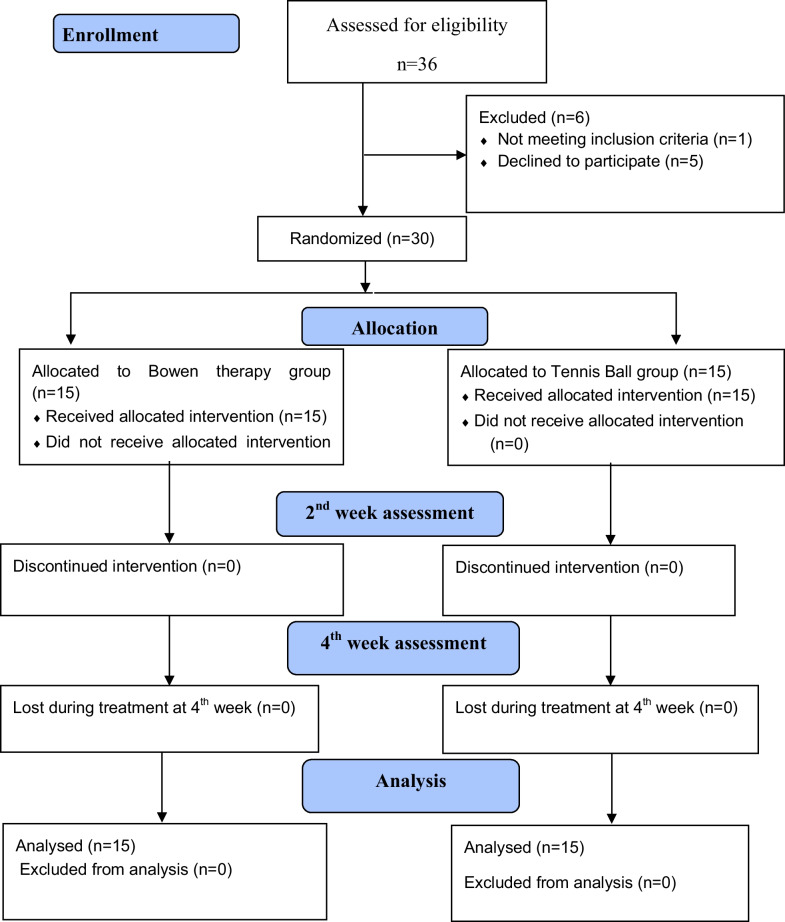
Consort diagram

Patients were asked to place the tennis ball between the thoracic region along medial border of scapula and wall or floor under the supervision of therapist. Both sides of spine were treated. Then they were asked to apply specific pressure to the aching spot along the medial border of scapula. After having relax period of 2 min, pressure was reapplied. The session last for 15 min with three repetitions [14]. Three treatment sessions were given in a week for total four weeks. Mid-treatment assessment was done on second week and follow-up assessment was on fourth week. Figure 1 is indicating CONSORT flow diagram.

### Sample size

Sample size was calculated by online EPITOOL keeping 95% confidence interval and 80% power and by putting the post-treatment pain values of Numeric Pain Rating Scale. After adding 10%, attrition rate sample size was 30 patients (15 in each group) [21].

### Data analysis

Version 26 of SPSS for Windows was used to analyze the data. The statistical significance was set at p ≤ 0.05. The tests utilized were as follows:

### Descriptive statistics

Frequency tables were utilized to display an overview of the group measures taken throughout time. For each variable, the mean and standard deviation were computed. The Shapiro–Wilk test was used to examine the assumptions of normality, and the results showed no obvious violations (*p* = 0.054–2.00). Analysis of variance (ANOVA) was performed to assess the differences within group after adjusting for pre-test scores in order to determine whether group had the more successful intervention. The 95% confidence interval was kept when the *p*-value was less than 0.05, indicating that the result was significant. Between the two groups, the difference in effect size was assessed by Cohen’s d. Effect sizes of 0.2, > 0.5, and > 0.8, respectively, were categorized as small, medium, and large.

## Results

Table 1 The mean value of age, weight, height, and BMI of participants in group A was 28.87 ± 4.87, 71.47 ± 10.19, 1.71 ± 0.048, 24.44 ± 2.83 and the mean value of age, weight, height, and BMI of participants in group B was 30.67 ± 4.53, 76.67 ± 7.93, 1.71 ± 0.064, 26.08 ± 1.96. The mean value of age, weight, height, and BMI of total participants was 29.77 ± 4.71, 74.07 ± 9.35, 1.71 ± 9.35, 25.26 ± 2.53. The NPRS mean value at baseline was 6.60 ± 0.63 in group A, and the NPRS mean value at baseline was 6.53 ± 0.63 in group B and *p* > 0.05 showed there is no difference between groups at baseline. The PDQ mean value at baseline was 91.87 ± 6.03 in group A and PDQ mean value at baseline was 91.40 ± 4.21 in group B and *p* > 0.05 showed there is no difference between groups at baseline.

**Table 1:** Demographic and baseline characteristics of participants

Demographic variables	Group A Mean ± SD	Group B Mean ± SD	Total (n = 30)	*p*-Value
Age	28.87 ± 4.87	30.67 ± 4.53	29.77 ± 4.71	0.303
Weight	71.47 ± 10.19	76.67 ± 7.93	74.07 ± 9.35	0.130
Height	1.71 ± 0.048	1.71 ± 0.064	1.71 ± 0.057	0.796
BMI	24.44 ± 2.83	26.08 ± 1.96	25.26 ± 2.53	0.077
NPRS	6.60 ± 0.63	6.53 ± 0.63	6.57 ± 0.63	0.776
PDQ	91.87 ± 6.03	91.40 ± 4.21	91.63 ± 5.12	0.808

*BMI* Body mass index, *NPRS* Numeric Pain Rating Scale, *PDQ* Pain Disability Questionnaire

The normality of data was assessed by Shapiro–Wilk test. The data were found to be normally distributed (P = 0.127 to0.265); therefore, parametric test was applied.

Table 2 shows that mean value of NPRS at baseline was 6.60 ± 0.63, 4.20 ± 0.68 at second week and 1.73 ± 0.703 at fourth week in group A. The results revealed that NPRS mean in the group B at baseline was6.53 ± 0.63, 5.20 ± 0.68 at second week and 2.87 ± 0.64 at fourth week. The mean difference between groups at second week was 1.000, and the mean difference between groups comparison at fourth week was 1.133. P-value was 0.000, showing significance. Effect size was large at second week and fourth week showed clinical significance.

**Table 2:** Between-group analysis for PDQ and NPRS

Follow-ups	Group A	Group B	Effect size	Mean difference 95% CI	*p* Value
	Mean ± SD	Mean ± SD			
NPRS (2nd week)	4.20 ± 0.68	5.20 ± 0.68	1.4	1.00 (1.505–0.4943	0.000
NPRS (4th week)	1.73 ± 0.703	2.87 ± 0.64	1.6	1.13 (1.636–0.6303)	0.000
PDQ (2nd week)	76.87 ± 3.54	82.333 ± 3.7	1.5	5.5 (8.1745–2.758)	0.000
PDQ (4th week)	63.07 ± 3.2	72.13 ± 3.5	2.7	9.1 (11.574–6.559	0.000

Table 3 is indicating the within group differences. There was a significant decrease in PDQ scores from the first to the third measurement. There were significant reductions in NPRS scores from the initial measurement to the following ones, indicating that both treatments were successful in reducing pain over time. Group A had larger mean differences, suggesting it was potentially more effective.

**Table 3:** Within-group analysis for NPRS and PDQ

Within Group change	Group A		Group B	
	Mean difference (95% CI)	*p* Value	Mean difference (95% CI)	*p* Value
NPRS				
Baseline	2.4 (1.819–2.981)	0.000	1.33 (0.991–1.676)	0.000
2nd week	4.87 (2.981–1.819)	0.000	3.67 (2.982–4.351)	0.000
4th week	2.47 (2.018–2.916)	0.000	2.33 (1.553–3.114)	0.000
PDQ				
Baseline	15 (10.68–19.33)	0.000	9.4 (7.17–1.63)	0.000
2nd week	28.8 (24.08–33.52)	0.000	19.6 (15.97–23.24)	0.000
4th week	13.8 (10.09–17.51)	0.000	10.2 (6.87–13.53)	0.000

## Discussions

The recent study was conducted to campare the effects of Bowen therapy and tennis ball technique on pain and functional disabillity in patients with myofascial pain syndrome. Thirty participants were selected randomly after meeting the inclusion and exclusion criteria. Within-group analysis indicated that participants receiving Bowen therapy and tennis ball technique showed a statistically significant reduction in pain, and improvements in functional disability with *p*-value < 0.05. Although both the therapies were found to be effective, Bowen therapy showed better results as compared to tennis ball technique. Mechanism of action of Bowen therapy is that it stimulates the sensory fibers of nervous system which in turn helps to restore body’s health [22]. Tennis ball is a myofascial release technique that provide flexibility to muscle by reducing adhesions in fibrous tissue of muscle [23]. The findings of the present study are consistent with those of an earlier study in which Bowen therapy showed significant results. It was a randomized controlled experiment in which group A received a Bowen therapy intervention, while group B received Muscle Energy Technique (METs) treatment. According to that study's findings, the group that used the Bowent treatment improved more than the other group in terms of hamstring muscle's flexibility, range of motion, and strength. However, the findings were similar but outcomes used and the length of treatment given was different from the present study [24]. Another study compared the effectiveness of Bowen therapy and METs in treating text neck syndrome. Group A received METs coupled with a hot pack, while group B received Bowen therapy. Results demonstrated the values of Bowen therapy in reducing pain and enhancing range of motion and functional status. Comparing participants treated with METs alone to those treated with Bowen therapy and METs together revealed substantial gains in functional status and pain reduction. So it ws concluded that Bowen therapy can be used as an adjunct to physiotherapy treatment with other intervention. These results are constant with the results of current study which showed Bowen therapy is more effective treatment approach [25].

The outcomes of this study are consistent with earlier investigations of patients with dyspraxia, a developmental problem. Boys with dyspraxia aged 8 to 11 were the subjects of this study. An eight-week treatment period was followed by a post-treatment evaluation. The study's findings demonstrated the positive effects of Bowen therapy on motor function. Following the fascia treatment with Bowen therapy, motor function significantly improved [26]. In contrast a research conducted on chronic pain. The effects of Bowen therapy were discovered through a study done on patients with persistent pain. Participants were split into an actual group and a fictitious group. The groups had eight-week therapy sessions. A sham treatment group was contrasted with the Bowen therapy group. After one week of treatment, the real group experienced decreased discomfort. However, during the follow-up, there was no difference in functional status between the two groups. Although it was determined that Bowen therapy had a quick or immediate impact but towards the end of the treatment protocol's eighth week, there was no sigficance between two groups noted [20]. Another study was in contrast of recent study which showed short-term effect of bowen therapy. It was conducted on postural neck pain. This study looked at how well patients with postural neck pain responded to the Bowen treatment when combined with conventional physiotherapy to lessen pain and enhance function. For reducing neck impairment caused on by postural neck discomfort in dentists, the Bowen treatment has been found to be just as effective as conventional therapy. At the end of the course of treatment, it was determined that there was little difference between these regimens [27]. The results of this investigation are consistent with those of the earlier study, which demonstrated the efficacy of Bowen therapy in treating myofascial pain syndrome. The participants had neck myofascial discomfort. It was a control trial. The experimental group underwent Bowen therapy whereas the control group received standard care. The progress in pain intensity and cervical range of motion was greater in the experimental group. Following the use of Bowen therapy, the scores of additional variables also decreased. Comparative study revealed that Bowen therapy was more successful at reducing pain and enhancing functional status [28]. The result of another study is in comparison with the recent study, according to which Bowen therapy led to a statistically significant improvement in the health of the patients. This previous study was conducted to show the efficacy of the Bowen technique in treating generalized lumbar spine pain disorders. Results of study conculded that the initial treatment led to the highest improvement in pain, functional disability and the range of motion [29].

The outcomes of previous studies are consistent with the results of the recent investigation. These studies' conclusions concur with one another. Bowen treatment is a special method for enhancing functionality and reducing pain. For patients with myofascial pain syndrome, it can be used as an extra therapeutic option to enhance functional outcomes.

In 2020, Fariba Eslamian et al. studied the effects of electroacupuncture and biofeedback on neck and upper back myofascial pain syndrome. This randomized clinical trial showed that both treatment protocols were equally effective. Intergroup comparison showed that electroacupuncture was more effective in some parameters over biofeedback treatment [30]. In Malak et al. [29] studied the effects of Bowen therapy in low back patients. This study included fifty patients with non-specific lumbar spine pain syndrome. The results of this study showed improvement in patients' health due to Bowen therapy. The increase in range of motion of the lumbar spine was observed. It showed that there was significant improvement after the first treatment session. The result of Bowen therapy after the third assessment was very good. It was concluded that the number of patients who were very satisfied with the efficacy of the treatment increases from the procedure to the procedure.

## Conclusion

This study concluded that Bowen therapy produced statistically significant and clinically relevant results for all the outcome measures. Bowen therapy was proved to be more effective in reducing pain and improving functional disability as compared to tennis ball technique.

### Limitation and recommendation

There was lack of considering the other factors like depression and anxiety and the results cannot be generalized to all age groups. It is recommended that future research should conduct larger sclae randomized controlled trials to confirm the effectiveness of Bowen therapy and consider long-term follow-up to assess the sustained effects of Bowen therapy beyond the immediate intervention and study should also be conducted to see the effects of Bowen therapy on aged population.

## Data Availability

The datasets generated and analyzed during the present study are not publicly available due to limitations of ethical approval involving the patient data and anonymity but are available from the corresponding author on reasonable request.

## Abbreviations

BT: Bowen therapy
MPS: Myofascial pain syndrome
NPRS: Numeric Pain Rating Scale
PDQ: Pain Disability Questionnaire
